# Premature aging of circulating T cells predicts all-cause mortality in hemodialysis patients

**DOI:** 10.1186/s12882-020-01920-8

**Published:** 2020-07-13

**Authors:** Fangfang Xiang, Rongyi Chen, Xuesen Cao, Bo Shen, Xiaohong Chen, Xiaoqiang Ding, Jianzhou Zou

**Affiliations:** 1 Department of Nephrology, Zhongshan Hospital, Fudan University, NO180, Feng’lin Road, Xuhui District, Shanghai, 200032 P.R. China; 2 Shanghai Key Laboratory of Kidney and Blood Purification, Shanghai, China; 3 Shanghai Institute of Kidney and Dialysis, Shanghai, China; 4 Shanghai Medical Center for Kidney, Shanghai, China

**Keywords:** Hemodialysis, T cell aging, naïve T cells, Mortality, Inflammation

## Abstract

**Background:**

Patients with end-stage renal disease (ESRD) exhibit a premature aging phenotype of immune system, which is recently concerned as a significant factor for increased risk of various morbidities. Nevertheless, there are few dates explicating the relevancy of T cell senescence to mortality. In this study, we prospectively studied the predictive value of T cell senescence for mortality in hemodialysis patients.

**Methods:**

Patients who had been on hemodialysis treatment for at least 6 months were enrolled. T cell senescence determined by differentiation status was evaluated by flow cytometry. Survival outcomes were estimated using the Kaplan-Meier method. Univariate and multivariate analyses were performed to evaluate the prognostic impact of T cell premature aging and other clinical factors on all-cause mortality.

**Results:**

A total of 466 patients (277 man and 169 women) were enrolled in this study. Decreased number of naïve T cell, as the most prominent feature of T cell senescence, did not change in parallel with age in these patients. Decreased absolute count of T cell, naïve T cell, CD4^+^ naïve T cell were independently associated with all-cause mortality. Decreased percentage of T cell and increased percentage of CD8^+^central-memory T cell were also independently associated with all-cause mortality. After including all the T cell parameters in one regression model, only decreased count of naïve T cell was significantly associated with increased mortality in these patients.

**Conclusions:**

Aging-associated T cell changes are aggravated in ESRD patients. For the first time, our study demonstrates that naïve T cell depletion is a strong predictor of all-cause mortality in HD patients.

## Background

End-stage renal disease (ESRD) patients suffer from much higher mortality compared to chronological age-matched individuals, mainly due to a high risk of cardiovascular disease (CVD) and infections [1], which also prevails in elderly people. Actually, besides CVD and infection, much more aging-related complications are increased in ESRD patients including malnutrition, impaired physical function, muscle wasting, cognitive function decline and malignancies [2, 3]. This proposes the hypothesis that uremia induces premature senescence, and chronic kidney disease (CKD) may be a model of premature aging. The decline of the immune system with age is thought to be the core factor behind these manifestations since the immune system is not only involved in controlling infections and malignancies, but also in tissue homeostasis and repair [4, 5]. Focusing on aging immune system, therefore, is a priority with crucial implications to reveal pathogenic mechanism of chronic diseases such as renal failure and to improve outcomes for these patients.

Age-related changes in the immune system, which are generally referred to as immunosenescence, are well documented and concern primarily the adaptive immune responses [4]. Among which, altered T cell function has been the most dramatic and consistent change reported during aging. In this content, T cells should be a very good spot to shed light into the uremia-related immune alteration. Recent evidence suggests uremia-related immune changes resemble to aging immune system, increasing immunological age of T cells by 20–30 years [6, 7]. As compared to an age-matched healthy control, ESRD patients present a lower thymic output of naïve T cells, a decline in the T-cell telomere length and an increase in the differentiation status towards the terminal differentiated memory phenotype with a large number of CD28-negative T cells. More importantly, these changes are strongly associated with CVD and occurrence of severe infectious episodes in ESRD patients [8, 9], supporting the idea that premature senescence in T cell compartment is a critical feature in this population and will impact clinical outcomes profoundly.

Although uremic associated T cell premature aging is well documented and concerned with a significant factor for increased risk of various morbidities; there is few dates explicating the relevancy of T cell senescence to mortality. This study prospectively researched the predictive value of T cell senescence determined by differentiation status for all-cause mortality and provided reference values for both absolute number and percentage of these T cell parameters in each decade of life within hemodialysis (HD) patients.

## Methods

### Study populations

This current study included HD patients who had been on HD treatment for at least 6 months in Blood Purification Center, Department of Nephrology, Zhongshan Hospital, Fudan University. Patients were enrolled from august to September in 2016 and followed until death, transfer to other clinic, renal transplantation, peritoneal dialysis, or July 15, 2019. Exclusion criteria were HD patients with recent or current infections, hematological diseases, rheumatic diseases, active malignancies, history of human immunodeficiency virus infection or currently use of any immunosuppressants. For all patients met the inclusion criteria, the clinical datas were recorded, including age, gender, body mass index (BMI), smoking behavior, blood pressure, underlying kidney disease and comorbidities such as hypertension, diabetes and CVD. CVD were defined as cardiac, cerebrovascular, or peripheral vascular disease.

All methods were carried out in accordance with relevant guidelines and regulations. This study was approved by the Ethical Committee, Zhongshan Hospital, Fudan University, and all the patients provided written informed consent.

### Cells preparation

Blood samples were drawn from the arterial site of the vascular access at the start of each dialysis session after a 2-day interval and stored in heparin tubules (BD Biosciences, San Diego, CA). Blood samples were lysed with red blood cell lysis solution [10 mM KHCO3, 155 mM NH4Cl (Sangon Biotech) and 0.1 mM EDTA (Sigma- Aldrich, St Louis, MO, USA)]. Subsequently, the cells were washed twice and resuspended in staining buffer containing PBS with 0.2% FBS (Invitrogen, Grand Island, NY, USA) and 0.09% NaN3 (Sigma-Aldrich).

### Flow cytometry analysis

Lysed cells were acquired and subsequently stained for 30 min at 4 °C with the following fluorescein-conjugated monoclonal antibodies: CD3-PE (Bio- Legend, San Diego, CA, USA), CD4-APC (eBioscience, San Diego, CA, USA), CD8a-Percp/Cy5.5 (eBioscience), CD45RO-FITC (Miltenyi Biotec, Bergisch Gladbach, Germany) and CCR7-APC/Cy7 (BioLegend). The T cell subsets were defined as previous study reported [10]: Naive T cells being CCR7^+^ and CD45RO^−^; central memory T cells as CD45RO^+^ and CCR7^+^; effector memory T cells as CD45RO^+^ and CCR7^−^, and effector memory RA (EMRA) T cells as CD45RO^−^ and CCR7^−^ (Figure S1). A total of 200,000 events were acquired by the BD LSRFortessa™ flow cytometer (BD Bioscience, San Jose, CA, USA). The data analysis was carried out with Flowjo v10.1 Software (Tree Star, Ashland, OR). The absolute number of each T cell subset was calculated as follows: (percentage of each cell population among total lymphocytes) × (total lymphocytes count)/100.

### Statistical analysis

All data were expressed as mean ± SD or median (interquartile range) appropriately. To analysis the correlation between T cell parameters and age, One-way ANOVA and linear analysis were used. Survival was estimated using the Kaplan–Meier curve and differences were examined using the log-rank test. Univariate Cox regression analysis was used to identify predictors of total death. Significant predictors were subsequently added to the multivariable model and backward stepwise Cox regression identified the most parsimonious model. The probability used for the stepwise regression was set at 0.05 for entry of variables and 0.1 for removal. Results of the Cox proportional hazards analysis were presented as the hazard ratio and the 95% confidence interval. *P* value of < 0.05 was considered statistically significant. All statistical calculations were performed with SPSS version 20.0 (SPSS Inc., Chicago, IL, USA).

## Results

### Patients characteristics

A total of 446 patients (277 man and 169 women) were enrolled in this study. The average age of patients was 59.3 ± 14.4 years. The median time on HD was for 54 (28, 83) months. The underlying kidney diseases were composed of chronic glomerulonephritis (47.5%), diabetic nephropathy (16.8%), polycystic kidney disease (8.7%), hypertension renal disease (3.1%), others (11.4%), and unknown (12.3%). Of the 466 patients, 103 (23.1%) had diabetes mellitus and 395 (79.1%) had hypertension. 141 patients (31.6%) had CVD history, of which 45 had more than one CVD complication. CVD incidents included 7 myocardial infarctions, 15 angina pectoris, 60 congestive heart failures, 64 cerebral infarctions, 10 cerebral hemorrhages and 9 peripheral vascular diseases. Only 3 out of 446 ESRD patients were seronegative for CMV (99.3% seropositive). Table 1 presents baseline characteristics of the study population.

**Table 1:** Demographic data of the study population

Variable	mean ± SD /median (interquartile range)
Age, years	59.3 ± 14.4
Time on HD, months	54(28,83)
Male, (%)	276(61.7%)
Diabetes mellitus, (%)	103(23.1%)
CVD history, (%)	141(31.6%)
Hypertension, (%)	395(79.1%)
CMV seropositive, (%)	443(99.3%)
BMI (kg/m^2^)	21.5 ± 3.2
Kt/Vurea	1.32 ± 0.56
Hemoglobin, g/L	112.1 ± 16.3
White blood cell, ×10^9/L	6.50 ± 2.00
Lymphocytes, ×10^9/L	1.3 ± 0.5
Albumin, g/L	39.0 ± 3.2
Prealbumin, g/L	0.32 ± 0.13
Creatinine, μmol/L	1005.5 ± 278.8
Uric acid, mmol/L	441.4 ± 87.5
Calcium, mmol/L	2.32 ± 0.25
Phosphorus, mmol/L	2.17 ± 0.64
Total cholesterol, mmol/L	4.11 ± 1.06
Triglyceride, mmol/L	1.45(1.03,2.23)
LDL-C, mmol/L	2.27 ± 0.86
HDL-C, mmol/L	1.06 ± 0.58
Homocysteine, μmol/L	34.8(26.5,46.7)
NT-proBNP, pg/ml	3859.0(1805.3, 10,384.0)
iPTH, pg/ml	261.2(150.7, 425.6)
Ferritin, pg/mL	293.2(129.3490.8)
hsCRP, mg/L	3.8(1.4,10.0)

*CVD* cardiovascular disease; *CMV* cytomegalovirus; *BMI* Body mass index; *LDL-C* low density lipoprotein -cholesterol; *HDL-C* high density lipoprotein- cholesterol; *NT-proBNP* N-terminal pro-brain natriuretic peptide; *hsCRP* high sensitivity-C reactive protein; *iPTH* intact parathyroid hormone

### Comparison of T cell senescence in different age period among hemodialysis patients

**Fig. 1:**
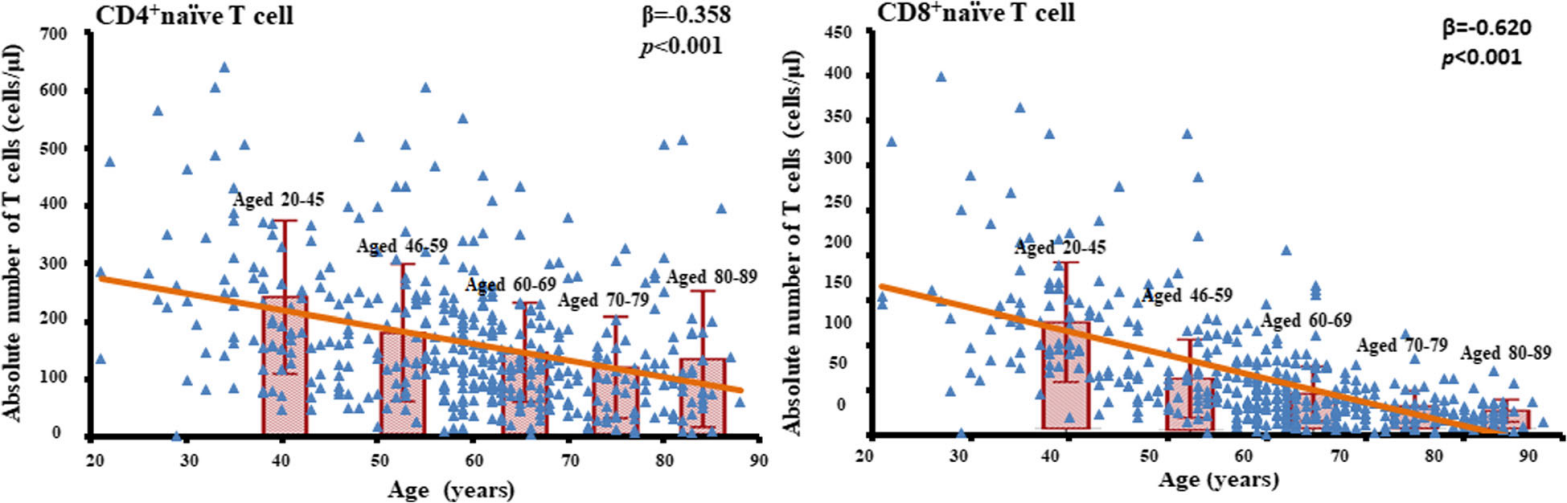
Correlations between naïve T cells and age. Scatter plots and regression lines demonstrated the relationship between T cell parameters with age in ESRD patients. Linear regression analysis showed that both CD4^+^ and CD8^+^ naïve T cell counts were negatively correlated to age. After dividing patients into 5 groups according to age period, CD4^+^ naïve T cell count decreased significantly with age in patients aged from 20 to 69 years old. Afterwards, there was no significant difference in CD4^+^ naïve T cell count, and even a little increase in 80–89 years old. CD8^+^ naïve T cell count decreased significantly with age in patients aged from 20 to 89 years old

To analyze the correlation between T cell parameters and age, patients were divided into 5 groups according to age. T cell parameters were expressed as median (interquartile range), as shown in Table 2. There was a significant difference in absolute number of CD4^+^T cells, CD4^+^naïve T cells, CD4^+^EMRA T cells, CD8^+^T cells, CD8^+^ naïve T cells among 5 groups (*p* < 0.05). However, these T subsets did not decrease in parallel with age. In the pairwise comparison, the absolute number of CD4^+^T cells decreased significantly with age in patients aged from 20 to 69 years old. Afterwards, there was no significant difference and even a little increase in 80–89 years old. This was mainly due to the changes of CD4^+^ naïve T cells, since they showed the same trend. The absolute number of CD4^+^ EMRA T cells was significantly higher in the 20–45 age group than other groups, and there was no significant difference in older age groups. A similar kinetics was observed in CD8^+^ T cell number, with a significant decrease with age in patients aged from 20 to 59 years old. CD8^+^ naïve T cells decreased significantly with age in a nearly parallel pattern in patients aged from 20 to 89 years old. It was presumed that CD8^+^ memory T cells expanded after 60 years old so that number of CD8^+^ T cells pools was relative remained, although statistical difference was not shown in these memory T cell parameters. Correlations between naïve T cells and age were shown in Fig. 1. When came to percentages, there was a significant difference in almost all the T cell parameters across age groups except CD4^+^ EMRA T cells. Linear analysis indicated percentages of naïve T cells were negatively correlated and percentages of memory T cells were positively correlated with age in both CD4^+^ and CD8^+^ compartment (*p* < 0.001).

**Table 2:** values of percentages and absolute numbers of T cell subset during aging

	ALL	Aged 20–45	Aged 46–59	Aged 60–69	Aged 70–79	Aged 80–89	*P* value
	*N* = 446	*N* = 84	*N* = 128	*N* = 129	*N* = 63	*N* = 42	
**Cell subset percentage**							
CD4^+^T cells %*	57.1(49.6,64.6)	55.5(49.2,60.4)	58.2 (52.3,67.0)	57.9(50.4,65.8)	54.6(47.6,63.2)	56.2(48.3,67.0)	0.02
CD4^+^ TNAIVE %*	36.7(26.2,46.8)	43.5(34.0,53.1)	37.1(26.5,48.3)	34.9(26.2,45.5)	29.5(20.7,44.9)	32.9(20.1,45.3)	< 0.001
CD4^+^TCM%*	21.7(15.1,28.7)	17.0(12.4,25.7)	21.3(15.2,25.9)	23.5(15.4,31.2)	23.1(18.5,31.4)	22.6(15.3,29.4)	0.002
CD4^+^TEM%*	33.2(25.4,42.3)	28.7(22.3,34.8)	32.8(25.4,42.0)	35.1(25.2,43.3)	37.9(26.2,45.6)	36.1(26.1,45.6)	< 0.001
CD4^+^TEMRA%	4.2(2.5,7.6)	4.8(2.8,8.9)	4.9(2.8,7.5)	4.3(2.5,7.9)	3.4(2.2,6.3)	3.6(2.0,6.2)	0.932
CD8^+^T cells %*	34.6(28.5,41.3)	36.2(32.3,42.1)	32.7(26.9,40.3)	33.2(26.8,39.4)	36.1(30.3,46.3)	36.0(27.5,43.9)	0.003
CD8^+^ TNAIVE % *	19.9(12.2,30.3)	37.1(28.4,52.5)	22.7(16.5,33.1)	15.5(13.0,24.9)	10.5(7.2,17.7)	9.5(5.3,15.4)	< 0.001
CD8^+^ TCM%*	2.8(1.7,4.5)	2.1(1.4,2.9)	2.7(1.7,4.3)	3.5(2.0,4.9)	3.1(1.7,4.9)	3.1(1.7,5.3)	0.001
CD8 ^+^TEM%*	22.5(15.5,30.9)	17.3(12.6,23.1)	22.0(16.1,30.2)	25.5(16.0,33.0)	27.1(17.2,36.2)	24.7(16.2,38.4)	< 0.001
CD8^+^TEMRA%*	49.7(37.0,60.3)	40.1(27.9,50.0)	48.1(34.1,58.1)	52.2(39.7,59.9)	57.9(43.3,69.8)	63.1(45.8,70.4)	< 0.001
CD4^+^Tcell /CD8^+^ T cell	1.64(1.21,2.25)	1.50(1.18,1.88)	1.76(1.31,2.49)	1.73(1.31,2.54)	1.56(1.01,2.02)	1.52(1.10,2.43)	0.051
**Absolute cell number**							
CD4^+^T cells (cells/μl)*	387(291,548)	500(367,636)	428(326,549)	369(279,524)	352(211,447)	345(264,411)	< 0.001
CD4^+^ TNAIVE (cells/μl)*	135(81,224)	226(148,292)	151(86,232)	124(84,186)	101(41,147)	104(56,175)	< 0.001
CD4^+^TCM (cells/μl)	85(54,126)	86(50,140)	84(55,125)	88(55,125)	79(54,130)	80(47,112)	0.324
CD4^+^TEM (cells/μl)	129(86,187)	136(91,189)	139(88,190)	127(89,180)	115(83,172)	108(78,197)	0.487
CD4^+^TEMRA (cells/μl)*	18(10,32)	25(11,46)	20(10,36)	16(10,28)	13(6,28)	13(7,20)	0.005
CD8^+^T cells (cells/μl)*	236(162,355)	318(251,448)	226(163,353)	209(138,315)	217(159,326)	219(139,321)	< 0.001
CD8^+^ TNAIVE (cells/μl)*	44(23,83)	129(85,164)	54(35,83)	37(21,62)	25(16,38)	20(13,30)	< 0.001
CD8^+^ TCM (cells/μl)	5(3,8)	5(3,7)	5(3,8)	6(3,9)	6(3,10)	5(3,9)	0.363
CD8 ^+^TEM (cells/μl)	41(28,61)	41(32,53)	39(27,62)	41(26,63)	46(28,71)	39(29,64)	0.28
CD8^+^TEMRA (cells/μl)	111(63,181)	119(77,193)	103(58,185)	103(54,164)	121(83,189)	115(63,194)	0.281

Percentages and absolute numbers (cells/μl) of naïve (TNAIVE), central memory (TCM), effector memory (TEM), terminally differentiated (TEMRA)

*p for trend across age groups < 0.05

### Naïve T cell count as a predictor of all-cause mortality in hemodialysis patients

All the patients were followed weekly, and follow-up ended in July, 2019. The median follow-up was for 33 months (range, 1–34 months) corresponding to a total follow-up of 1049 patient-years. During follow-up, 103 patients died, 11 patients had renal transplantation, 2 were transferred to peritoneal dialysis and 23 were transferred to another clinic. The most common cause of mortality was cardiovascular death (death due to myocardial infarction, heart failure, cerebrovascular accident or peripheral vascular disease) (*n* = 54; 52.4%), followed by infection (*n* = 17; 16.5%), sudden death (*n* = 9; 8.7%), cancer (*n* = 7; 6.8%), gastrointestinal hemorrhage (*n* = 7; 6.8%) and others (*n* = 9; 8.7%).

**Fig. 2:**
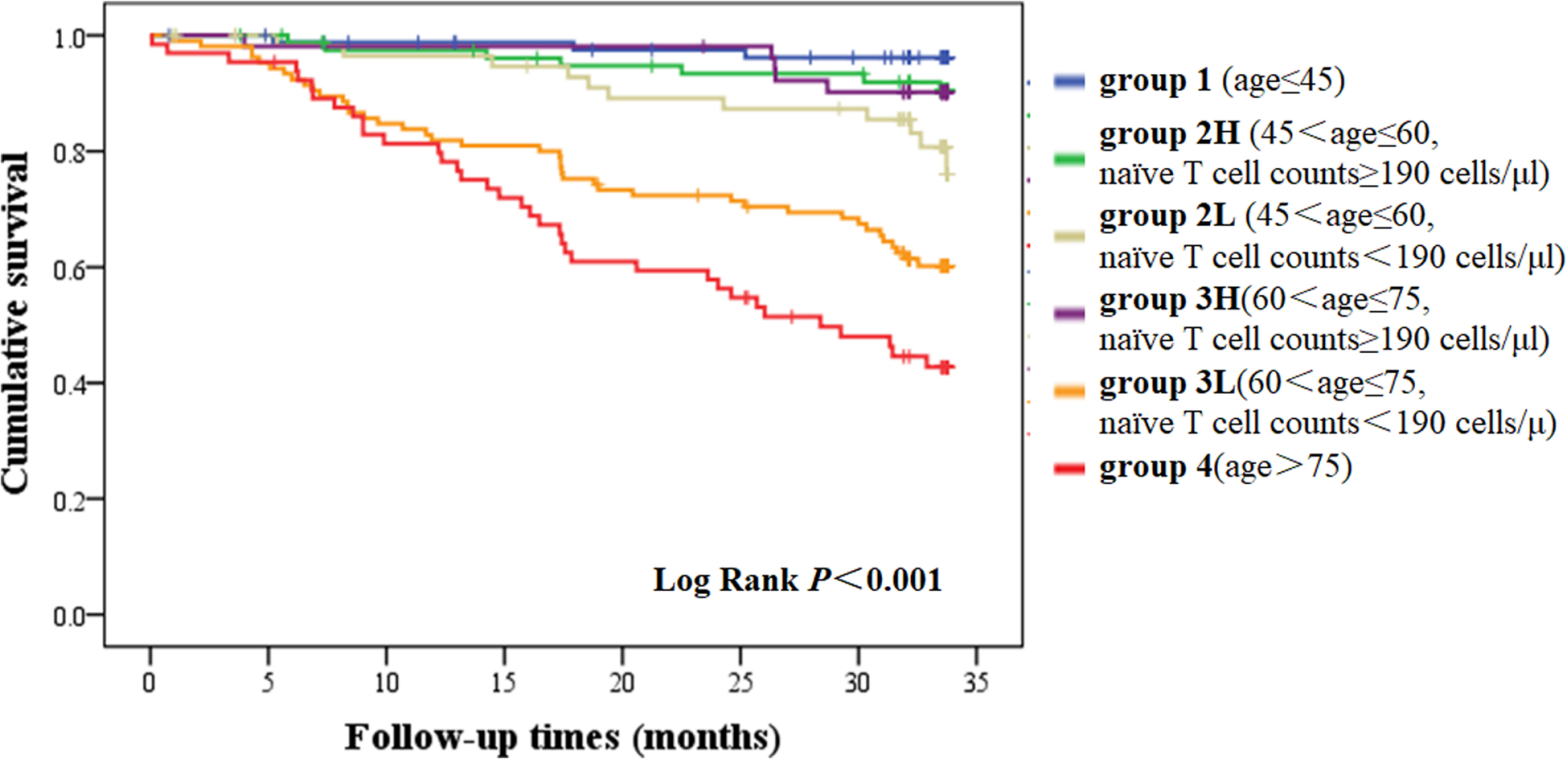
Overall survival curves according to age - naïve T group. Patients were divided into six groups according to age and value of naïve T cell count. Kaplan-Meier analysis revealed that survival rate was significantly different between six age-naïve T groups (*p* < 0.001). In pairwise comparison, survival rate was significantly lower in the oldest group when compared with other groups. Patients age 61–75 years old with a lower naïve T cell count had a significant lower survival rate than those in the same age period but with a higher naïve T cell count (*p* < 0.001). Patients aged 46–60 years old with a lower naïve T cell count also had a significant lower survival rate than those in the youngest age period (*p* = 0.04). Patients aged 46–75 years old with a higher naïve T cell count seemed to have a similar survival rate compared to patients younger than 46 years old

We divided the patients into six groups according to age and value of naïve T cell count. The median level of naïve T cell was 190 cells/μl. Group 1 included patients younger than 46 years old (*n* = 84). Group 2 L included patients aged 46 to 60 years old with naïve T cell count below the median level (*n* = 60). Group 2H included patients aged 46 to 60 years old with naïve T cell count above the median level (*n* = 80). Group 3 L included patients aged 61 to 75 years old with naïve T cell count below the median level (*n* = 105). Group 3H included patients aged 61 to 75 years old with naïve T cell count above the median level (*n* = 52). Group 4 included patients older than 75 years old (*n* = 65). Kaplan-Meier analysis revealed that survival rate was significantly different between six age-naïve T groups (*p* < 0.001). In pairwise comparison, survival rate was significantly lower in the oldest group when compared with other groups. Patients aged 61–75 years old with a lower naïve T cell count had a significant lower survival rate than those in the same age period but with a higher naïve T cell count (*p* < 0.001). Patients aged 46–60 years old with a lower naïve T cell count also had a significant lower survival rate than those in the youngest age period (*p* = 0.04). Notably, patients aged 46–75 years old with a higher naïve T cell count seemed to have a similar survival rate compared to patients younger than 46 years old (Fig. 2). CD4^+^ and CD8^+^ naïve T cell count showed a similar effect in predicting all-cause mortality in these patients (Figure S2, Figure S3).

In univariate cox proportional hazard model, decreased absolute count of T cells, naïve T cells, CD4^+^ naïve T cells, CD8^+^ naïve T cells were significant predictors of mortality. Decreased percentage of T cells, naïve T cells, CD4^+^ naïve T cells, CD8^+^ naïve T cells and increased percentage of CD4^+^effector-memory T cells, CD8^+^central-memory T cells, CD8^+^effector-memory T cells, CD8^+^EMRA T cells were also significant correlated with higher mortality rate. Other mortality predictors included older age, history of CVD, diabetes mellitus, lower serum level of albumin, prealbumin, urea nitrogen, creatinine, uric acid and increased serum level of high sensitivity-C reactive protein, soluble interleukin-2 receptor, N-terminal pro-brain natriuretic peptide (Table 3). After adjusted with various conventional and unconventional risk factors related to mortality, decreased absolute count of T cells, decreased absolute count of naïve T cells, decreased absolute count of CD4^+^ naïve T cells, decreased percentage of T cells and increased percentage of CD8^+^ central-memory T cells along with older age, history of diabetes, history of CVD, decreased albumin level and elevated NT-proBNP level were independently associated with all-cause mortality. After including all the significant T cell parameters in one regression model, only decreased count of naïve T cell was significantly associated with increased mortality in those patients (Table 4).

**Table 3:** Univariate Cox hazard model for all-cause mortality in hemodialysis patients

Variables	Hazard Ratio (95% Confidence Interval)	*P* value
Age (years)	1.070 (1.053, 1.088)	< 0.001
Sex (male = 1)	1.121 (0.751, 1.672)	0.577
Diabetes mellitus (yes = 1)	1.911 (1.271, 2.874)	0.002
CVD (yes = 1)	3.977 (2.679, 5.906)	< 0.001
BMI (kg/m^2^)	0.959 (0.907, 1.015)	0.149
Kt/Vurea	0.893 (0.586, 1.360)	0.598
Time on HD (month)	0.996 (0.992, 1.001)	0.086
Hemoglobin (g/L)	0.991 (0.979, 1.002)	0.119
Albumin (g/L)	0.816 (0.773, 0.862)	< 0.001
Prealbumin (g/L)	0.021 (0.003, 0.154)	< 0.001
Urea nitrogen (mmol/L)	0.965 (0.935, 0.995)	0.024
Creatinine (μmol/L)	0.998 (0.997, 0.999)	< 0.001
Uric acid (mmol/L)	0.995 (0.993, 0.998)	< 0.001
Phosphorus (mmol/L)	0.826 (0.606, 1.125)	0.225
Calcium (mmol/L)	0.602 (0.279, 1.296)	0.195
Log-iPTH (pg/ml)	0.973 (0.584, 1.619)	0.915
β2-Microglobulin (mg/L)	1.020 (0.995, 1.045)	0.117
Homocysteine (μmol/L)	0.993 (0.985, 1.001)	0.067
Log-hsCRP (mg/L)	1.930 (1.376, 2.705)	< 0.001
Log-sIL-2R (U/ml)	16.328 (4.157, 64.140)	< 0.001
Log-NT-proBNP (pg/mL)	4.089 (2.690, 6.216)	< 0.001
T cell count (cells/μl)	0.223 (0.113, 0.440)	< 0.001
Naïve t cell count (cells/μl)	0.002 (0.000, 0.018)	< 0.001
CD4^+^naïve T cell count (cells/μl)	0.001 (0.000, 0.012)	< 0.001
CD8^+^naïve T cell count (cells/μl)	0.000 (0.000, 0.000)	< 0.001
T cell (%)	0.059 (0.015, 0.241)	< 0.001
Naïve T cell (%)	0.032 (0.006, 0.159)	< 0.001
CD4^+^naïve T cell (%)	0.086 (0.023, 0.327)	< 0.001
CD4^+^effector-memory T cell (%)	8.563 (2.139, 34.271)	0.002
CD8^+^naïve T cell (%)	0.006 (0.001, 0.042)	< 0.001
CD8^+^central memory T cell (%)	2.313 (1.211, 4.421)	0.011
CD8^+^effector memory T cell (%)	7.253(1.684, 31.242)	0.008
CD8^+^EMRA T cell (%)	4.290 (1.367, 13.461)	0.013

*CVD* cardiovascular disease; *BMI* Body mass index; *HD* hemodialysis; *Log-hsCRP* log transformed high sensitivity-C reactive protein; *Log-sIL-2R* log transformed soluble interleukin-2 receptor; *Log-NT-proBNP* log transformed N-terminal pro-brain natriuretic peptide

**Table 4:** Multivariate Cox proportional hazard model for all-cause mortality

Variables	Model 1		Model 2		
	Hazard Ratio (95% CI)	*P* value	Hazard Ratio (95% CI)	*P* value	
T cell count (cells/μl)	0.325 (0.146, 0.719)	0.006			
Naïve T cell count (cells/μl)	0.042 (0.004, 0.429)	0.008	0.030 (0.004, 0.247)	0.001	
CD4^+^naïve T cell count (cells/μl)	0.031 (0.002, 0.496)	0.014			
CD8^+^naïve T cell count (cells/μl)	0.000 (0.000, 1.133)	0.053			
T cell (%)	0.080 (0.014, 0.445)	0.004			
CD8^+^central-memory T cell (%)	2.261 (1.092, 4.681)	0.028			
CD8^+^effector-memory T cell (%)	4.946 (0.849, 28.827)	0.075			
CD8^+^EMRA T cell (%)	0.251 (0.063, 1.008)	0.051			

Backward conditional method was used. Model 1 included each T cell parameters and was adjusted for age, sex, BMI, history of CVD, history of diabetes, dialysis duration, hemoglobin, albumin, prealbumin, urea nitrogen, creatinine, uric acid, phosphorus, calcium, intact parathyroid hormone, β2-microglobulin, homocysteine, soluble interleukin-2 receptor, N-terminal pro-brain natriuretic peptide and high-sensitivity C-reactive protein. Model 2 included all the related T cell parameters and was adjusted for the same factors as model 1

## Discussion

To the best of our knowledge, the present study has been the first one to evaluate differentiation status of peripheral T lymphocyte in predicting mortality in ESRD patients. The main finding was highlighted as follows: decreased naïve T cell is a strong predictor of all-cause mortality in HD patents.

In this study, we analyzed circulating T cell subsets of 466 ESRD patients for each decade of life. Our finding consisted with earlier studies that aging affected lymphocyte subpopulation profile of ESRD patients with a decrease of absolute numbers of naïve T cells and an increase of percentage of memory T cells [11, 12]. Decreased number of naïve T cell seems to be the most prominent phenomenon of T cell senescence, no matter it is caused by aging or ESRD. Chiu YL et al. posted a dramatic 40–50% reduction in CD4^+^ and CD8^+^ naïve T cell numbers in 412 ESRD patients when compared to age-matched healthy individuals [12]. Freitas et al. suggested that age and ESRD presented additive effect decreasing naïve T cells without synergic effect [11]. However, this study was based on a small sample size and patients were roughly divided into two groups if age was over 60 or not. In the current study, since we provided values for the absolute numbers and proportions of T cell subsets in each decade of life, it allowed us to analyze the trend of T cell variation with more detailed information. We found that the number of CD4^+^ naïve T cell didn’t change in parallel with age and patients over 80 years old seemed to have a relatively increased naïve T cells in CD4^+^ compartment, which was not reported in healthy elderl y[13]. Although there was no statistical difference owing to the sample size, it suggested that uremia and age might affect differently to immune system in extremely old ESRD patients.

In the analysis of predictors of mortality, we discovered naïve T cell depletion was independently associated with all-cause mortality. By analyzing the influence of physiological and immunological age, we found patients presented with lower naïve T cell count were associated with a significantly lower survival compared to those in the same age period but with a higher naïve T cell count. It led to consumption that naïve T cell could be a valuable marker of presenting overall immunological age in ESRD patients. In this respect, every patient should have this test as a part of immune disturbance evaluation and risk assessment of multiple complications, even with early intervention if possible. Another dominant effect by ESRD is a significant increase in percentage of memory T cells with advanced differentiation, which is also universal in age-related immune senescence. In this study, we find increased percentage of CD8^**+**^central-memory T cell was independently associated with mortality, which can be caused by increased naïve T cell turnover [14]. While increased percentage of CD8^+^EMRA T cell, on the other hand, could not predict mortality independently. This also confirmed that loss of naïve T cells might be a hallmark of immune aging, while increased differentiated T cells might partly if not entirely due to the decrease of naive T cells. This is partly explained by some epigenetic studies [15, 16].

Observations over the past 10 years have concordantly indicated that the process of naïve T cell homeostasis is profoundly affected by aging, with the changes aimed to maintain naïve T cell pool eventually leading to its further depletion and demise [17, 18]. Several factors need to be considered to grasp both the quantitative and qualitative changes of the naive T cells with aging:their production by the thymus, their homeostatic proliferation and the diversity of their TCR repertoire, which are all disturbed in uremic setting. A decreased thymic output of naïve T cells was observed in both CD4 and CD8 compartment in ESRD patients and was associated with severe infection episode and cardiovascular events in these patients [6, 9]. Data of thymic function are lacking for ESRD patient. However, it was reported that renal failure could lead to involution of thymus and spleen in animal experiments [19, 20]. A recent study indicated serum PTH concentrations were related to thymus atrophy evaluated by recent thymic emigrants, indicating mineral and bone disorder might be an underling mechanism [21]. Besides thymus atrophy, malfunction of peripheral maintenance may be a profounder reason of marked decreased naïve T cell in ESRD patient. Interleukin-7, as the central factor in maintaining naïve T cells, does not decline with age but decreases in ESRD patients [22, 23], indicating there may be a relatively insufficient increase in homeostatic proliferation in these patients. However, impaired proliferation function is not the major case in naïve T cell senescence. Actually, most studies support age-related loss of naive T cells in both healthy individuals and ESRD patients associated with an increased turnover [6, 23–25]. The most significant factor associated with T cell turnover is inflammation. It is posted that excess cytokines and increased expression of cytokine receptors, such as those produced in inflammatory conditions, could be detrimental for homeostasis and accelerate immune aging [26, 27]. Accordingly, accelerated aging has been described in several autoimmune diseases [28, 29]. In ESRD patients, inflammation is significantly enhanced by uremia [30], which could theoretically accelerate the processes of aging. Chronic immune stimulation could also lead to clonally expanded T cell population [31, 32]. Recent studies indicated ESRD patients presented reduced T-cell receptor diversity with clonal expansions [33, 34], which may has several clinical implications as it may increase the risk for infections, malignancies and CVD [14]. In addition, TCR-mediated signaling was also disturbed among these patients [35].

CMV infection has a substantial impact on the composition and function of circulating T cells and is recognized increasingly as a significant factor for T cell aging [36]. In infected humans, CMV induces an inflation of both the CD4^+^ and CD8^+^ effector-memory T cells, followed by a dramatic shrinkage of TCR repertoire, as such may add to the increased risk of infections as well as CVD in healthy and ESRD individuals [37, 38]. In ESRD, 70–100% patients are CMV seropositive [12, 39]. According to previous research, the additional effects of CMV latency on T cell ageing parameters in young to middle-aged ESRD patients were modest and confined mainly to the CD8^+^ T cells [40]. While in elderly ESRD patients, CMV latency seemed to promote highly differentiation in both CD4^+^ and CD8^+^ T cells [41]. In addition, recent studies indicated a higher level of CMV-IgG was associated with advanced T-cell differentiation and coronary artery disease [42]. It is also important to note that the expansion of CD28^**−**^ T cells seems only occurred in CMV-seropositive patients with ESRD [43], while CD28^−^ T cells had significant pro-inflammatory and cytotoxic function and was highly associated with cardiovascular complications, thus CMV infection should account for increased cardiovascular morbidity in CKD patients [38]. In this study, more than 99% patients were CMV seropositive, which may confound the effect to premature T cell ageing by CMV infection and uremia itself. However, since most studies indicated CMV infection mostly affected memory T cells, while in the current study we certainly found a strong association between naïve T cells rather than memory T cells with mortality, therefore CMV may not be a major reason of unpleasant prognosis in these patients.

Other factors could also lead to immune senescence in patients with CKD. Notably, decreased kidney function per se and the uremic milieu affect most of the factors known to accelerate aging, including DNA damage, phosphate toxicity, klotho deficiency, oxidative stress and telomere shortening [2]. Accumulation of uremic toxins is another important factor inducing premature aging of the T cells [9, 12, 23]. In addition, the treatment recipe including hemodialysis and iron supplementation may further exacerbate immunological ageing of the T cell compartment in these patients [44, 45]. After renal transplantation, despite of pro-inflammatory cytokines and oxidative stress decreasing to normal levels, uremia- associated prematurely aged T-cell immune system still exists [46], indicating uremia associated T cell aging may not be reversible. Deeper mechanistic insight into the phenomena of premature ageing as well as early diagnosis of CKD might improve the application and efficacy of interventions and provide novel lead to combat CVD and infection in CKD.

Our study had several limitations. First, since the study population was composed of 99% CMV seropositive patients, it was not known if the findings could be extrapolated to CMV seronegative ESRD patients. Secondly, T cells may exhibit aging-related changes in their effector functions that were not reflected by phenotypic changes. Finally, this was a single-center study, which might potentially limit the statistical power and its external validity.

## Conclusions

In conclusion, this study posted that naïve T cells depletion was a strong predictor of total mortality in HD patients. Assessing T-cell ageing parameters could be useful for picturing the whole immune function and early identifying patients at high risk of profound complications. Since many factors of maintaining naïve T cells are disturbed in ESRD patients, further researches are required to promulgate the underling mechanism and explore effective method of preventing or even reversing uremia associated T cell premature aging.

## Supplementary information

**Additional file 1.** Figure S1. Flowchart of flow cytometry analysis to identify T cell subset. T cell subsets were defined by flow cytometry: Naive T cells as CCR7+ and CD45RO-; central memory T cells as CD45RO+ and CCR7+; effector memory T cells as CD45RO+ and CCR7-, and EMRA T cells as CD45RO- and CCR7-.

**Additional file 2 **Figure S2. Overall survival curves according to age – CD4^+^naïve T group. Patients were divided into six groups according to age and value of CD4^+^ naïve T cell count. Kaplan-Meier analysis revealed that survival rate was significantly different between six age- CD4^+^ naïve T groups (*p* < 0.001).

**Additional file 3 **Figure S3. Overall survival curves according to age – CD8^+^naïve T group. Patients were divided into six groups according to age and value of CD8^+^ naïve T cell count. Kaplan-Meier analysis revealed that survival rate was significantly different between six age- CD8^+^ naïve T groups (*p* < 0.001).

## Data Availability

The datasets supporting the current study are available from the corresponding author on reasonable request.

## Abbreviations

BMI: Body mass index
CKD: Chronic kidney disease
CM: Central memory
CMV: Cytomegalovirus
CVD: Cardiovascular disease
ESRD: End-stage renal disease
EM: Effector memory
EMRA: Effector memory RA
HD: Hemodialysis
HDL-C: High density lipoprotein- cholesterol
hsCRP: high sensitivity-C reactive protein
LDL-C: Low density lipoprotein -cholesterol
NT-proBNP: N-terminal pro-brain natriuretic peptide
iPTH: intact parathyroid hormone
sIL-2R: soluble interleukin-2 receptor

## References

[1] Kooman JP, Kotanko P, Schols AM, Shiels PG, Stenvinkel P (2014). Chronic kidney disease and premature ageing. NAT REV NEPHROL.

[2] Stenvinkel P, Larsson TE (2013). Chronic kidney disease: a clinical model of premature aging. Am J Kidney Dis.

[3] Chiu YL, Tsai HH, Lai YJ, Tseng HY, Wu YW, Peng YS, Chiu CM, Chuang YF (2019). Cognitive impairment in patients with end-stage renal disease: accelerated brain aging?. J Formos Med Assoc.

[4] Goronzy JJ, Weyand CM (2013). Understanding immunosenescence to improve responses to vaccines. Nat Immunol.

[5] Fülöp T, Dupuis G, Witkowski JM, Larbi A (2016). The Role of Immunosenescence in the Development of Age-Related Diseases. Revista de investigacion clinica.

[6] Betjes MG, Langerak AW, van der Spek A, de Wit EA, Litjens NH (2011). Premature aging of circulating T cells in patients with end-stage renal disease. Kidney Int.

[7] Betjes MG (2013). Immune cell dysfunction and inflammation in end-stage renal disease. NAT REV NEPHROL.

[8] Betjes MGH, de Wit EEA, Weimar W, Litjens NHR (2010). Circulating pro-inflammatory CD4posCD28null T cells are independently associated with cardiovascular disease in ESRD patients. Nephrol , Dial Transpl.

[9] Crépin T, Legendre M, Carron C, Vachey C, Courivaud C, Rebibou J, Ferrand C, Laheurte C, Vauchy C, Gaiffe E, et al. Uraemia-induced immune senescence and clinical outcomes in chronic kidney disease patients. Nephrol Dial Transpl. 2020;35(4):624–32. 10.1093/ndt/gfy276 30202981

[10] Ruud WMMG (2014). T-cell ageing in end-stage renal disease patients: assessment and clinical relevance. World J Nephrol.

[11] Freitas GRR, Da Luz FM, Agena F, Jaluul O, Silva SC, Lemos FBC, Coelho V, Elias D, Galante NZ (2019). Aging and end stage renal disease cause a decrease in absolute circulating lymphocyte counts with a shift to a memory profile and diverge in Treg population. Aging Dis.

[12] Chiu Y, Shu K, Yang F, Chou T, Chen P, Lay F, Pan S, Lin C, Litjens NHR, Betjes MGH (2018). A comprehensive characterization of aggravated aging-related changes in T lymphocytes and monocytes in end-stage renal disease: the iESRD study. Immun Ageing.

[13] Provinciali M, Moresi R, Donnini A, Lisa RM (2009). Reference values for CD4+ and CD8+ T lymphocytes with Naïve or memory phenotype and their association with mortality in the elderly. GERONTOLOGY.

[14] Nikolich-Zugich J (2014). Aging of the T cell compartment in mice and humans: from no naive expectations to foggy memories. J Immunol.

[15] Moskowitz DM, Zhang DW, Hu B, Le Saux S, Yanes RE, Ye Z, Buenrostro JD, Weyand CM, Greenleaf WJ, Goronzy JJ. Epigenomics of human CD8 T cell differentiation and aging. Sci Immunol. 2017;2(8):eaag0192. 10.1126/sciimmunol.aag0192 PMC5399889 28439570

[16] Sen DR, Kaminski J, Barnitz RA, Kurachi M, Gerdemann U, Yates KB, Tsao HW, Godec J, LaFleur MW, Brown FD (2016). The epigenetic landscape of T cell exhaustion. Science.

[17] Goronzy JJ, Weyand CM (2017). Successful and maladaptive T cell aging. IMMUNITY.

[18] Goronzy JJ, Fang F, Cavanagh MM, Qi Q, Weyand CM (2015). Naive T cell maintenance and function in human aging. J Immunol.

[19] Levine S, Saltzman A (2001). Are urea and creatinine uremic toxins in the rat?. Ren Fail.

[20] Raskova J, Czerwinski DK, Shea SM, Raska JK (1986). Cellular immunity and lymphocyte populations in developing uremia in the rat. J Exp Pathol.

[21] Iio K, Kabata D, Iio R, Imai Y, Hatanaka M, Omori H, Hoshida Y, Saeki Y, Shintani A, Hamano T (2019). Parathyroid hormone and premature thymus ageing in patients with chronic kidney disease. SCI REP UK.

[22] Fry TJ, Mackall CL (2005). The many faces of IL-7: from Lymphopoiesis to peripheral T cell maintenance. J Immunol.

[23] Litjens NHR, van Druningen CJ, Betjes MGH (2006). Progressive loss of renal function is associated with activation and depletion of naive T lymphocytes. Clin Immunol.

[24] Tsukamoto H, Clise-Dwyer K, Huston GE, Duso DK, Buck AL, Johnson LL, Haynes L, Swain SL (2009). Age-associated increase in lifespan of Naïve CD4 T cells contributes to T-cell homeostasis but facilitates development of functional defects. P NATL ACAD SCI USA.

[25] Čičin-Šain L, Messaoudi I, Park B, Currier N, Planer S, Fischer M, Tackitt S, Nikolich-Žugich D, Legasse A, Axthelm MK (2007). Dramatic Increase in Naïve T Cell Turnover Is Linked to Loss of Naïve T Cells from Old Primates. P NATL ACAD SCI USA.

[26] López-Otín C, Blasco MA, Partridge L, Serrano M, Kroemer G (2013). The hallmarks of aging. CELL.

[27] Cheng T, Rodrigues N, Shen H, Yang Y, Dombkowski D, Sykes M, Scadden DT (2000). Hematopoietic stem cell quiescence maintained by p21cip1/waf1. SCIENCE.

[28] Goronzy JJ, Li G, Yang Z, Weyand CM (2013). The janus head of T cell aging - autoimmunity and immunodeficiency. Front Immunol.

[29] Goronzy JJ, Weyand CM (2012). Immune aging and autoimmunity. Cell Mol Life Sci.

[30] Stenvinkel P (2002). Inflammation in end-stage renal failure: could it be treated?. Nephrol Dial Transplant.

[31] Posnett DN, Sinha R, Kabak S, Russo C (1994). Clonal populations of T cells in normal elderly humans: the T cell equivalent to "benign monoclonal gammapathy". J Exp Med.

[32] Goronzy JJ, Bartz-Bazzanella P, Hu W, Jendro MC, Walser-Kuntz DR, Weyand CM (1994). Dominant clonotypes in the repertoire of peripheral CD4+ T cells in rheumatoid arthritis. J Clin Invest.

[33] Crawford DC, Bailey J, Miskimen K, Miron P, McCauley JL, Sedor JR, OToole JF, Bush WS (2018). Somatic T-cell receptor diversity in a chronic kidney disease PatientPopulation linked to electronic health records. AMIA Jt Summits Transl Sci Proc.

[34] Huang L, Betjes MGH, Klepper M, Langerak AW, Baan CC, Litjens NHR (2017). End-stage renal disease causes skewing in the TCR Vβ-repertoire primarily within CD8 + T cell subsets. Front Immunol.

[35] Huang L, Litjens N, Kannegieter NM, Klepper M, Baan CC, Betjes M (2017). pERK-dependent defective TCR-mediated activation of CD4(+) T cells in end-stage renal disease patients. Immun Ageing.

[36] Fülöp T, Larbi A, Pawelec G (2013). Human T cell aging and the impact of persistent viral infections. Front Immunol.

[37] Blankenberg S, Rupprecht HJ, Bickel C, Espinola-Klein C, Rippin G, Hafner G, Ossendorf M, Steinhagen K, Meyer J (2001). Cytomegalovirus infection with interleukin-6 response predicts cardiac mortality in patients with coronary artery disease. Circulation.

[38] Betjes MG, Litjens NH, Zietse R (2007). Seropositivity for cytomegalovirus in patients with end-stage renal disease is strongly associated with atherosclerotic disease. Nephrol Dial Transplant.

[39] Litjens N, Wit E, Betjes M (2011). Differential effects of age, cytomegalovirus-seropositivity and end-stage renal disease (ESRD) on circulating T lymphocyte subsets. Immun Ageing.

[40] Meijers RW, Litjens NH, de Wit EA, Langerak AW, van der Spek A, Baan CC, Weimar W, Betjes MG (2013). Cytomegalovirus contributes partly to uraemia-associated premature immunological ageing of the T cell compartment. Clin Exp Immunol.

[41] Huang L, Langerak AW, Baan CC, Litjens NH, Betjes MG (2016). Latency for cytomegalovirus impacts T cell ageing significantly in elderly end-stage renal disease patients. Clin Exp Immunol.

[42] Yang F, Shu K, Chen H, Chen I, Lay F, Chuang Y, Wu C, Tsai W, Peng Y, Hsu S (2018). Anti-cytomegalovirus IgG antibody titer is positively associated with advanced T cell differentiation and coronary artery disease in end-stage renal disease. Immun Ageing.

[43] Betjes MG, Huisman M, Weimar W, Litjens NH (2008). Expansion of cytolytic CD4+CD28- T cells in end-stage renal disease. Kidney Int.

[44] Meijers R, Litjens N, Wit E, Langerak A, Spek A, Baan C, Weimar W, Betjes M (2012). Uremia causes premature ageing of the T cell compartment in end-stage renal disease patients. Immun Ageing.

[45] Ducloux D, Legendre M, Bamoulid J, Rebibou JM, Saas P, Courivaud C, Crepin T (2018). ESRD-associated immune phenotype depends on dialysis modality and iron status: clinical implications. Immun Ageing.

[46] Meijers RWJ, Litjens NHR, Wit EA, Langerak AW, Baan CC, Betjes MGH (2014). Uremia-associated immunological aging is stably imprinted in the T-cell system and not reversed by kidney transplantation. Transpl Int.

