# Supplementary figures and images for: Premature aging of circulating T cells predicts all-cause mortality in hemodialysis patients
Source: BMC Nephrol. 2020 Jul 13;21:271. doi: 10.1186/s12882-020-01920-8 (PMC7359274; doi:10.1186/s12882-020-01920-8)

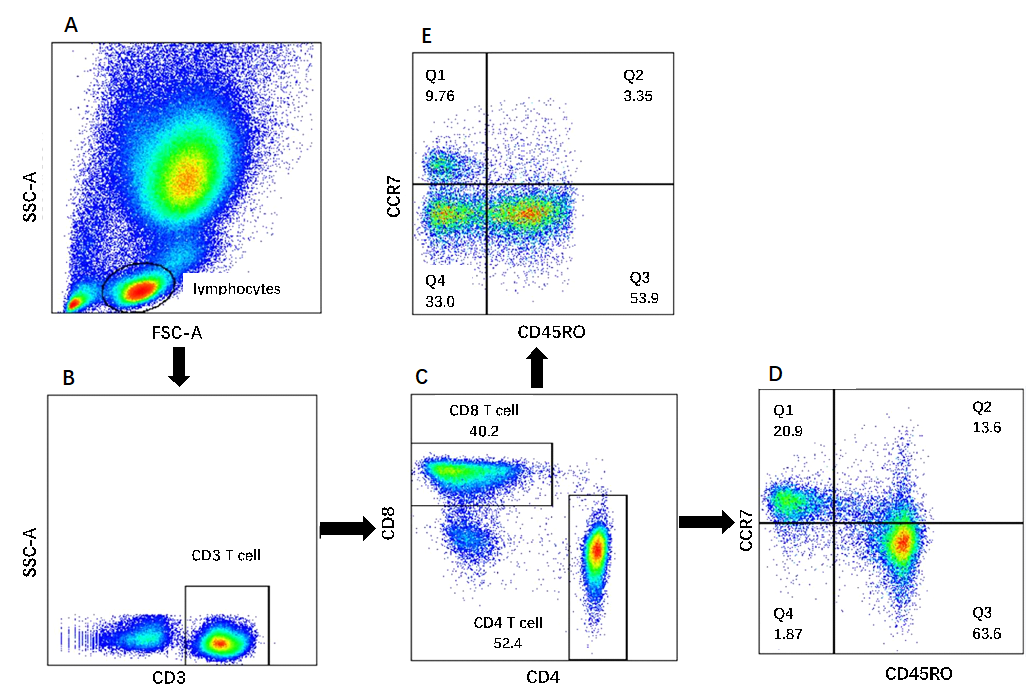

Supplement: Supplementary file 1 — Additional file 1. Figure S1. Flowchart of flow cytometry analysis to identify T cell subset. T cell subsets were defined by flow cytometry: Naive T cells as CCR7+ and CD45RO-; central memory T cells as CD45RO+ and CCR7+; effector memory T cells as CD45RO+ and CCR7-, and EMRA T cells as CD45RO- and CCR7-. [file 12882_2020_1920_MOESM1_ESM.tif]

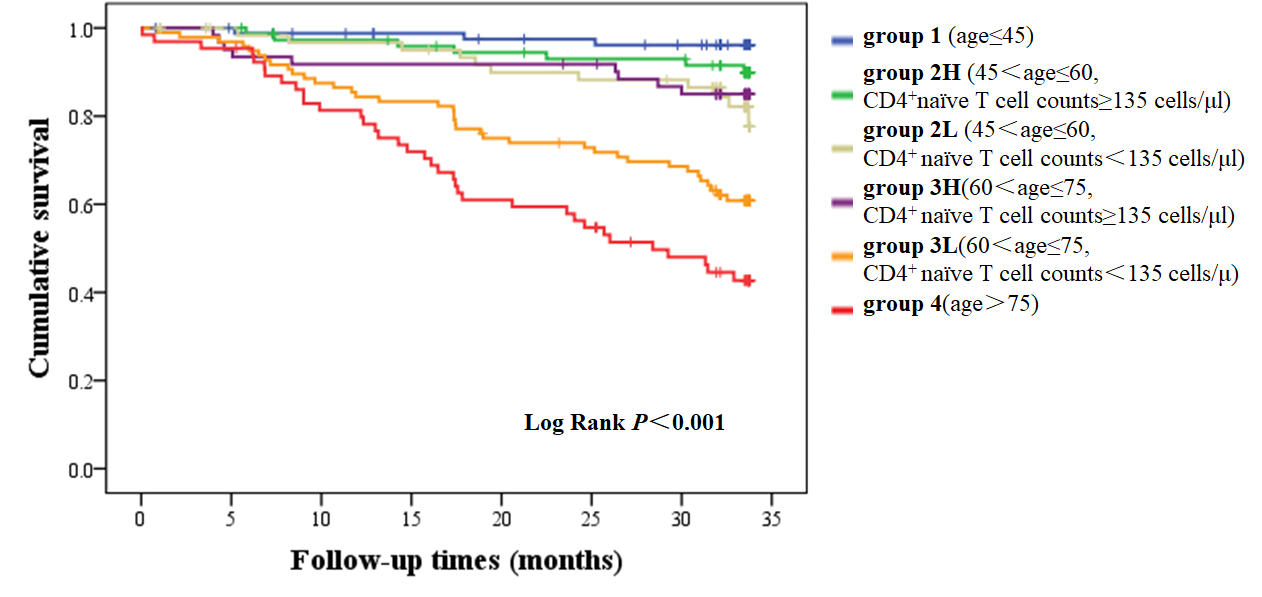

Supplement: Supplementary file 2 — Additional file 2 Figure S2. Overall survival curves according to age – CD4+naïve T group. Patients were divided into six groups according to age and value of CD4+ naïve T cell count. Kaplan-Meier analysis revealed that survival rate was significantly different between six age- CD4+ naïve T groups (p < 0.001). [file 12882_2020_1920_MOESM2_ESM.tif]

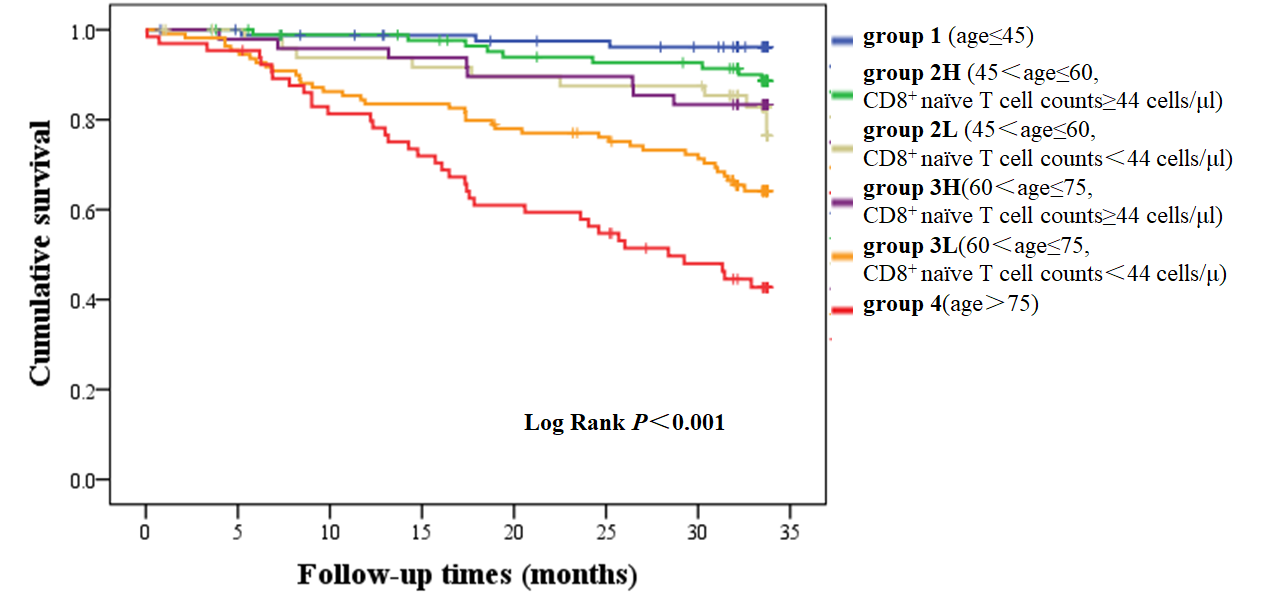

Supplement: Supplementary file 3 — Additional file 3 Figure S3. Overall survival curves according to age – CD8+naïve T group. Patients were divided into six groups according to age and value of CD8+ naïve T cell count. Kaplan-Meier analysis revealed that survival rate was significantly different between six age- CD8+ naïve T groups (p < 0.001). [file 12882_2020_1920_MOESM3_ESM.tif]
